# Association between fat amount of dairy products with pulse wave velocity and carotid intima-media thickness in adults

**DOI:** 10.1186/1475-2891-13-37

**Published:** 2014-04-24

**Authors:** Jose I Recio-Rodriguez, Manuel A Gomez-Marcos, Maria-C Patino-Alonso, Alvaro Sanchez, Cristina Agudo-Conde, Jose A Maderuelo-Fernandez, Luis Garcia-Ortiz

**Affiliations:** 1 La Alamedilla Health Centre, Castilla y León Health Service–SACYL, redIAPP, IBSAL, Salamanca, Spain; 2 Statistics Department, University of Salamanca, Salamanca, Spain; 3 Primary Care Research Unit of Bizkaia, Basque Health Service-Osakidetza, Bilbao, Spain; 4 Unidad de Investigación, Centro de Salud La Alamedilla, Avda. Comuneros 27-31, 37003 Salamanca, Spain

**Keywords:** Dairy products, Diet, Fat-restricted, Atherosclerosis, Pulse wave analysis, Carotid artery diseases

## Abstract

**Background:**

Examine the relation between consumption of low-fat vs. whole-fat dairy products with the carotid intima-media thickness and pulse wave velocity.

**Findings:**

Methods: Cross-sectional and multi-center study. A total of 265 subjects were selected by stratified random sampling. Measurements: Information about dairy products was assessed using a semi-quantitative food-frequency questionnaire. Carotid intima-media thickness (IMT) was measured by carotid ultrasonography. Pulse wave velocity (PWV) was measured using the SphygmoCor-System.

**Results:**

Subjects (age 55.8 ± 12.2) had mean values of IMT 0.68 ± 0.10 mm and PWV 7.60 ± 2.0 m/sec. The relationship between PWV and IMT with whole-fat and low-fat dairy intake groups, adjusted for age, sex, energy intake and other confounders revealed lower values of PWV in subjects with a consumption higher than 125 g/day of low-fat dairy and in those who did not intake whole-fat dairy. In a risk-factor adjusted regression model, an increase in PWV of 0.109 m/sec (95% CI: 0.006 –0.213) was estimated for every 100 g/day increase in whole-fat dairy intake. Similarly, a decrease in PWV of 0.101 m/sec (95% CI: −0.178 –0.023) was estimated for every 100 g/day increase in low-fat dairy intake, (p = 0.038 and p = 0.011 respectively). While for every 100 g/day increase in low-fat dairy intake, the estimate decrease of IMT was 0.005 mm (95% CI: −0.010 –0.001), p = 0.011.

**Conclusions:**

PWV and IMT showed an inverse association with the intake of low-fat dairy and a positive association with the intake of whole-fat dairy, so the amount of fat in dairy products can play an important role in arterial stiffness and subclinical atherosclerosis.

## Findings

### Background

Consumption of low-fat vs. whole-fat dairy products has been associated with a lower risk of stroke and coronary heart disease [1]. Recent studies have established a relationship between blood pressure and consumption of dairy products, in particular low-fat dairy, without finding association with other metabolic risk parameters [2]. Intima-media thickness of the common carotid artery (IMT) and pulse wave velocity (PWV) are intermediate risk markers in the development of cardiovascular disease [3,4]. Crichton et al. concluded that a higher intake of dairy products is associated with lower values of PWV [5]. Along the same lines, a recent study found significant benefits for the carotid intima-media thickness for greater consumption of yogurt [6]. However, the association of these parameters with the fat component of dairy products has not been previously described. The present study was done to explore the relationship between consumption of whole-fat vs. low-fat dairy and the vascular structure and function assessed by the IMT and PWV.

## Methods

### Study design and population

Subjects selected for this work come from the EVIDENT study and were selected by stratified random sampling. The EVIDENT study is a cross-sectional and multi-center study in which six groups participated from throughout Spain. The protocol was previously published [7]. Each general practitioner who collaborated in the study, selected, among all those who attended the clinic for any reason, these subjects, ages 20–80, who met the inclusion criteria and none of the exclusion criteria [7]. The results presented in this manuscript corresponded to the 265 subjects included in the Salamanca Health Center, the only center where the IMT and PWV were measured. This number of subjects was sufficient to detect a difference in the IMT of 0.05 mm between the three groups established according to their dairy products intake, assuming a SD of 0.1 with a significance level of 95% and a power of 80%. The study was approved by an independent ethic committee of Salamanca University Hospital (Spain), and all participants gave written informed consent according to the general recommendations of the Declaration of Helsinki. The recruitment and data collection was conducted along the years 2011 and 2012.

### Variables and measurement instruments

The dietary habits of participants and information about dairy products were derived from a previously validated semi-quantitative 137-item food frequency questionnaire (FFQ) collected at the time of the interview [8]. The FFQ included 137 food items, and the frequencies of consumption of the food items were reported on an incremental scale with nine levels (never or almost never, 1–3 times per month, once per week, 2–4 times per week, 5–6 times per week, once per d, 2–3 times per d, 4–6 times per d and more than six times per day. The reported frequencies of food consumption were converted to number of intakes per d and multiplied by the weight of the portion size indicated. The questionnaire was based on the typical portion sizes that were multiplied by the the consumption frequency for each food. Carotid ultrasonography to assess intima-media thickness of the common carotid artery (IMT) was performed with the Sonosite Micromax ultrasound device (Sonosite Inc., Bothell, Washington, USA) paired with a 5–10 MHz multifrequency high-resolution linear transducer. SonoCalc™ IMT software (version 3.4) was used for performing automatic measurements of IMT in order to optimize reproducibility. SonoCalc IMT is a tool that measures automatically the intima media thickness of the carotid artery and plaque using digital ultrasound images. SonoCalc IMT generates a report with the patient’s IMT value. This information can be used with other medical information to assess the cardiovascular health of a patient. Six measurements were taken on each carotid artery, using mean values (average IMT) and maximum values (maximum IMT) calculated by the software automatically. Measurements were taken following the recommendations of the Manheim Carotid Intima-Media Thickness Consensus [9]. Two investigators, whom were trained before starting the study, performed the IMT. The reliability of which was evaluated before the study began using the intraclass correlation coefficient, which showed values of 0.974 (95% CI: 0.935–0.990) for intra-observer agreement on repeated measurements in 20 subjects, and 0.897 (95% CI: 0.740–0.959) for inter-observer agreement. According to the Bland-Altman analysis, the limit of inter-observer agreement was 0.022 (95% CI: −0.053 to 0.098) and the limit of intra-observer agreement was 0.012 (95% CI: −0.034 to 0.059). Carotid-femoral pulse wave velocity (PWV) was estimated using the SphygmoCor System (AtCor Medical Pty Ltd., Head Office, West Ryde, Australia). PWV was measured with the patient in the supine position, estimating the delay in pulse wave at carotid and femoral level as compared to the electrocardiogram wave, according to the expert consensus document on arterial stiffness by Van Bortel et al. [10]. One investigator, whom was trained for this prior to starting the study, performed pulse wave velocity. The reliability of which was evaluated before the study began using the intraclass correlation coefficient, which showed values of 0.907 (95% CI: 0.786–0.960) for intra-observer agreement on repeated measurements in 25 subjects. According to the Bland-Altman analysis, the limit of inter-observer agreement was −0.079 (95% CI: −2.839 to 2.680). Further details on the EVIDENT study design have been published elsewhere [7].

### Statistical analysis

Continuous variables were expressed as mean ± standard deviation and frequency distribution was used in categorical variables. To analyze the relationship of the IMT and PWV with different groups according to their low-fat and whole-fat dairy intake (we considered 125 g/day as a typical portion of yogurt or half serving of milk), an analysis of the covariance (ANCOVA) was performed. In addition, we conducted two multiple linear regression models considering as dependent variables the IMT and the PWV and independent variables the consumption of low-fat and whole-fat dairy (g/day). To facilitate the interpretation of the results, in the multiple linear regression models, the IMT and the PWV have been multiplied by 100. Several authors have proposed that the patient’s age, sex, blood pressure and the presence of obesity, diabetes and vascular drugs, are the main determinants of the PWV and the IMT [11-13]. BMI and total energy intake may be influenced by the total consumption of dairy products. For all these reasons, we included the most important determinants of the PWV and the IMT and variables related to the consumption of dairy products as adjustment variables (age, sex, body mass index, smoking, energy intake, office or clinical systolic blood pressure, total cholesterol and the presence of diabetes and vascular drugs (antihypertensive, antidiabetic and lipid-lowering drugs)). Data were analyzed using IBM® SPSS® v.20 software. A value of p < 0.05 was considered statistically significant.

## Results

The demographic and clinical characteristics of participants (age 55.8 ± 12.2) are shown in Table 1, including the mean values of IMT (0.68 ± 0.10 mm) and PWV (7.60 ± 2.0 m/sec).

**Table 1:** Baseline patient characteristics

Age (years)	55.85 ± 12.21
Males (n, %)	107 (40.7)
Hypertension (n, %)	75 (28.5)
Diabetes (n, %)	17 (6.5)
Dyslipidemia (n, %)	72 (27.4)
Smoking (n, %)	54 (20.5)
BMI (kg/m^2^)	27.29 ± 4.31
Office systolic blood pressure. (mmHg)	122.3 ± 17.9
Office diastolic blood pressure. (mmHg)	77.6 ± 10.8
Antihypertensive drugs (n, %)	76 (28.9)
Lipid-lowering drugs (n, %)	45 (17.1)
Antidiabetic drugs (n, %)	12 (4.6)
Total cholesterol (mg/dL)	212.6 ± 37.9
LDL-cholesterol (mg/dL)	132.2 ± 33.4
Mean IMT (mm)	0.68 ± 0.10
PWV (m/sec)	7.60 ± 2.00
Energy intake (Kcal/day)	2623.6 ± 671.48
Whole-fat dairy (g/day)	102.2 ± 169.6
Low-fat dairy (g/day)	164.1 ± 222.9

Continuous variables are expressed as mean ± standard deviation and frequency distribution was used in categorical variables.

BMI: body mass index; IMT: Intima Media Thickness; PWV: pulse wave velocity.

**Figure 1:**
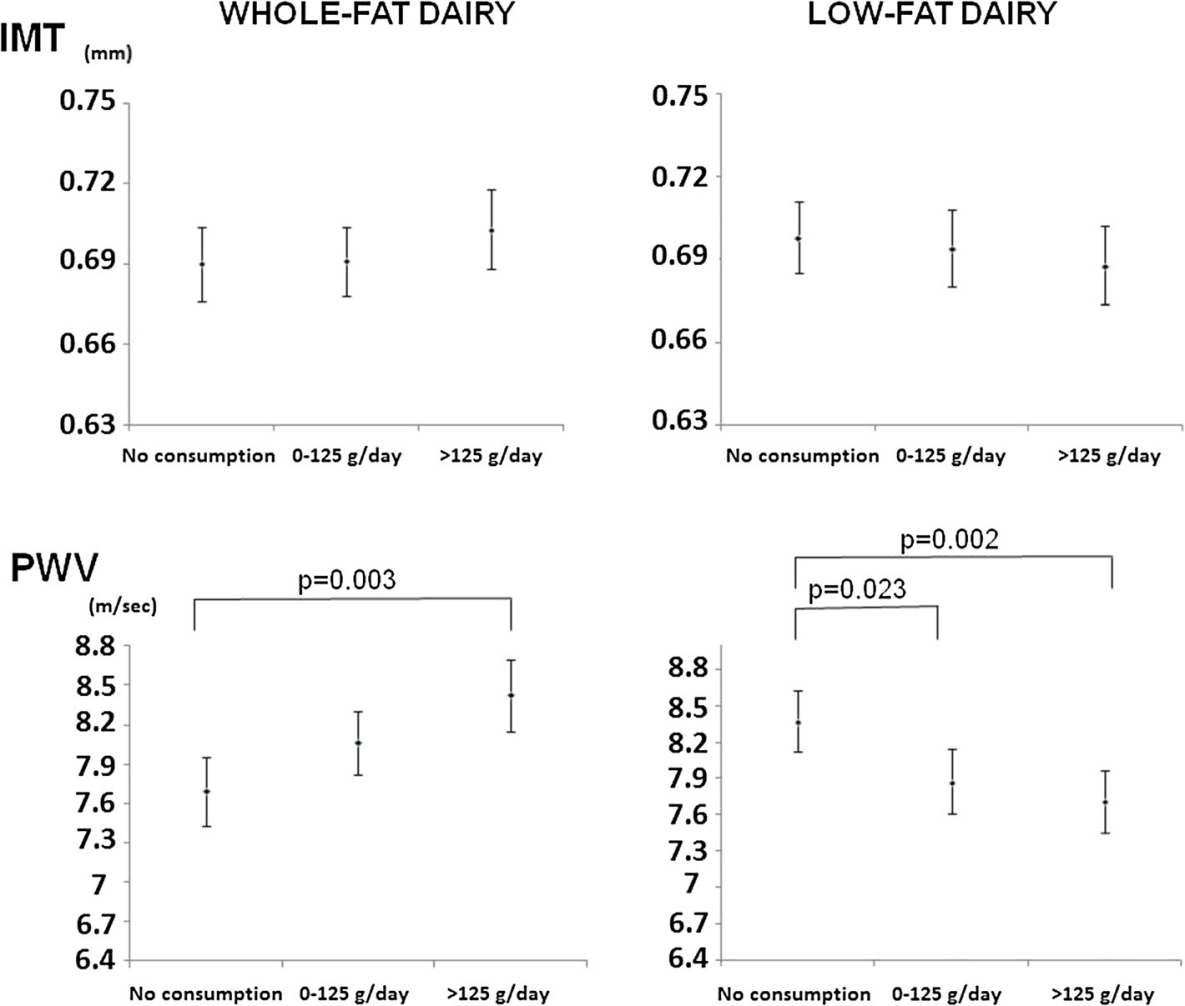
**Mean values of IMT and PWV in low-fat and whole-fat dairy intake groups after adjustment for age, sex, BMI, smoking, systolic blood pressure, total cholesterol, energy intake and the presence of diabetes, antihypertensive, antidiabetic and lipid-lowering drugs.** PWV: Difference between nonconsumers and those with >125 g/day of whole-fat and low-fat dairy (p < 0.003 and p = 0.002, respectively). IMT: Difference between nonconsumers and those with >125 g/day of whole-fat and low-fat dairy (p = 0.334 and p = 0.388, respectively).

Figure 1 represents the relationship between PWV and IMT with whole-fat and low-fat dairy intake groups, adjusted for age, sex, smoking, energy intake, BMI, systolic blood pressure, total cholesterol and the presence of diabetes, antihypertensive, antidiabetic and lipid-lowering drugs. Mean values of PWV were lower in subjects with a level of consumption higher than 125 g/day of low-fat dairy (7.71 m/sec) and in those who did not intake whole-fat dairy (7.69 m/sec), p = 0.002 and 0.003 respectively. In the same way, IMT showed a tendency towards reduction among those in the highest intake of low-fat dairy (0.688 mm) and those in the lowest intake of whole-fat dairy (0.690 mm), p > 0.05 both.

Based on a risk factor adjusted regression model, an increase in PWV of 0.109 m/sec (95% CI: 0.006 –0.213) was estimated for every 100 g/day increase in whole-fat dairy intake. Similarly, a decrease in PWV of 0.101 m/sec (95% CI: −0.178 –0.023) was estimated for every 100 g/day increase in low-fat dairy intake, (p = 0.038 and p = 0.011 respectively). While for every 100 g/day increase in low-fat dairy intake, the estimate decrease of IMT was 0.005 mm (95% CI: −0.010 –0.001), p = 0.011.

## Discussion

Pulse wave velocity and the carotid intima-media thickness showed a positive association with the intake of whole-fat dairy and inversely with consumption of low-fat dairy. This finding provides new evidence of a possible association between the fat component of dairy products and values of IMT and PWV.

Crichton et al. [5] found lower values of PWV in subjects whose consumption of dairy products was greater than 5–6 times per week (mean value: 9.9 m/sec) with respect to those with a regular intake of 2–4 times per week. However, in our study we have analyzed the consumption of whole-fat and low-fat dairy separately. We found that a consumption higher than 125 g/day respect of a non-regularly consume low-fat dairy is significantly associated with lower PWV values. The relationship between PWV and consumption of whole-fat dairy shows a completely opposite trend, with higher values of PWV in subjects with an intake of whole-fat dairy greater than 125 g/day. The only nutritional difference between whole dairy and low-fat dairy products is exclusively based on its fat content, and consequently, its caloric value. The fat content of dairy products may affect the blood lipid profile and promote atherosclerosis and cardiovascular disease.

Ivey et al. [6] found that the consumption of >100 g yogurt/d, but not milk or cheese was associated with a significantly lower IMT in a risk-factor adjusted model included age, BMI, energy intake, physical activity, use of vascular medication, diabetes and smoking. In our work, there is a tendency to lower values of IMT in consumers of >125 g/day of low-fat dairy and in subjects who do not usually consume whole-fat dairy, showing in a risk-adjusted factor model, an inverse association between the consumption of low-fat and lower IMT values. Possible relative benefits of consuming low-fat dairy versus whole-fat dairy involve decreased saturated fatty acids in the dairy products. Saturated fat intake may influence in the development of atherosclerosis and in the incidence of cardiovascular disease.

Finally, our results support the findings of Warensjö et al. [14]. This study reports a higher proportion of the milk fat biomarker 17:0 in the phospholipid fraction in plasma to be inversely related to the risk of a first event of stroke. The authors hypothesize that estimated milk fat intake is associated with a lower risk of first event stroke.

The main limitation of this study is the cross-sectional design that prevents from establishing causal relationships between IMT, PWV and consumption of dairy products. Second, the assessment of dairy products were based mainly on food frequency questionnaires which were designed to assess habitual diet by asking about the frequency with limited food items, but not specifically developed to assess the dairy intake.

## Conclusions

Pulse wave velocity and intima-media thickness showed an inverse association with the intake of low-fat dairy products and a positive association with the intake of whole-fat dairy, so the fat component of dairy products can play an important role in arterial stiffness and subclinical atherosclerosis. This fact will have to be confirmed in prospective studies.

## Abbreviations

(IMT): Intima-media thickness of the common carotid artery; (PWV): Pulse wave velocity; (FFQ): Food frequency questionnaire.

## Competing interests

The authors declare that they have no competing interests.

## Authors’ contributions

JIR devised the study, designed the protocol, participated in fund raising and interpretation of results, prepared the manuscript draft and corrected the final version of the manuscript. MGM, AMF and AS participated in the study design, interpretation of results and manuscript review. CAC participated in the study design, data collection and manuscript review. MCP performed all statistical analyses, participated in the interpretation of results, and manuscript review. LGO participated in the protocol design, fund raising, analysis of results, and final review of the manuscript. Finally, all authors reviewed and approved the final version of the manuscript.

## References

[B1] Dalmeijer GW Struijk EA van der Schouw YT Soedamah-Muthu SS Verschuren WM Boer JM Geleijnse JM Beulens JW Dairy intake and coronary heart disease or stroke-A population-based cohort study Int J Cardiol 2012 167 925 929 22483419 10.1016/j.ijcard.2012.03.094

[B2] Soedamah-Muthu SS Verberne LD Ding EL Engberink MF Geleijnse JM Dairy consumption and incidence of hypertension: a dose–response meta-analysis of prospective cohort studies Hypertension 2012 60 5 1131 1137 10.1161/HYPERTENSIONAHA.112.195206 22987924

[B3] Mitchell GF Hwang SJ Vasan RS Larson MG Pencina MJ Hamburg NM Vita JA Levy D Benjamin EJ Arterial stiffness and cardiovascular events: the Framingham Heart Study Circulation 2010 121 4 505 511 10.1161/CIRCULATIONAHA.109.886655 20083680 PMC2836717

[B4] Polak JF Pencina MJ O’Leary DH D’Agostino RB Common carotid artery intima-media thickness progression as a predictor of stroke in multi-ethnic study of atherosclerosis Stroke 2011 42 11 3017 3021 10.1161/STROKEAHA.111.625186 21885840 PMC3202068

[B5] Crichton GE Elias MF Dore GA Abhayaratna WP Robbins MA Relations between dairy food intake and arterial stiffness: pulse wave velocity and pulse pressure Hypertension 2012 59 5 1044 1051 10.1161/HYPERTENSIONAHA.111.190017 22431583 PMC3341626

[B6] Ivey KL Lewis JR Hodgson JM Zhu K Dhaliwal SS Thompson PL Prince RL Association between yogurt, milk, and cheese consumption and common carotid artery intima-media thickness and cardiovascular disease risk factors in elderly women Am J Clin Nutr 2011 94 1 234 239 10.3945/ajcn.111.014159 21613553

[B7] Garcia-Ortiz L Recio-Rodriguez JI Martin-Cantera C Cabrejas-Sanchez A Gomez-Arranz A Gonzalez-Viejo N Iturregui-San Nicolas E Patino-Alonso MC Gomez-Marcos MA Physical exercise, fitness and dietary pattern and their relationship with circadian blood pressure pattern, augmentation index and endothelial dysfunction biological markers: EVIDENT study protocol BMC Public Health 2010 10 233 10.1186/1471-2458-10-233 20459634 PMC2881095

[B8] Fernandez-Ballart JD Pinol JL Zazpe I Corella D Carrasco P Toledo E Perez-Bauer M Martinez-Gonzalez MA Salas-Salvado J Martin-Moreno JM Relative validity of a semi-quantitative food-frequency questionnaire in an elderly Mediterranean population of Spain Br J Nutr 2010 103 12 1808 1816 10.1017/S0007114509993837 20102675

[B9] Touboul PJ Hennerici MG Meairs S Adams H Amarenco P Bornstein N Csiba L Desvarieux M Ebrahim S Fatar M Hernandez Hernandez R Jaff M Kownator S Prati P Rundek T Sitzer M Schminke U Tardif JC Taylor A Vicaut E Woo KS Zannad F Zureik M Mannheim carotid intima-media thickness consensus (2004–2006). An update on behalf of the Advisory Board of the 3rd and 4th Watching the Risk Symposium, 13th and 15th European Stroke Conferences, Mannheim, Germany, 2004, and Brussels, Belgium, 2006 Cerebrovasc Dis 2007 23 1 75 80 10.1159/000097034 17108679

[B10] Van Bortel LM Laurent S Boutouyrie P Chowienczyk P Cruickshank JK De Backer T Filipovsky J Huybrechts S Mattace-Raso FU Protogerou AD Schillaci G Segers P Vermeersch S Weber T Artery Society; European Society of Hypertension Working Group on Vascular Structure and Function; European Network for Noninvasive Investigation of Large Arteries Expert consensus document on the measurement of aortic stiffness in daily practice using carotid-femoral pulse wave velocity J Hypertens 2012 30 3 445 448 10.1097/HJH.0b013e32834fa8b0 22278144

[B11] Determinants of pulse wave velocity in healthy people and in the presence of cardiovascular risk factors: ‘establishing normal and reference values’ Eur Heart J 2010 31 19 2338 2350 20530030 10.1093/eurheartj/ehq165 PMC2948201

[B12] Baldassarre D Nyyssonen K Rauramaa R de Faire U Hamsten A Smit AJ Mannarino E Humphries SE Giral P Grossi E Veglia F Paoletti R Tremoli E IMPROVE study group Cross-sectional analysis of baseline data to identify the major determinants of carotid intima-media thickness in a European population: the IMPROVE study Eur Heart J 2010 31 5 614 622 10.1093/eurheartj/ehp496 19952003

[B13] Su TC Chien KL Jeng JS Chen MF Hsu HC Torng PL Sung FC Lee YT Age- and gender-associated determinants of carotid intima-media thickness: a community-based study J Atheroscler Thromb 2012 19 9 872 880 10.5551/jat.10728 22972311

[B14] Warensjo E Smedman A Stegmayr B Hallmans G Weinehall L Vessby B Johansson I Stroke and plasma markers of milk fat intake–a prospective nested case–control study Nutr J 2009 8 21 10.1186/1475-2891-8-21 19457271 PMC2689251

